# Optimization of the Imaged cIEF Method for Monitoring the Charge Heterogeneity of Antibody-Maytansine Conjugate

**DOI:** 10.1155/2023/8150143

**Published:** 2023-06-02

**Authors:** Ayat Abbood

**Affiliations:** Department of Medicinal Chemistry and Quality Control, Faculty of Pharmacy, Tishreen University, Lattakia, Syria

## Abstract

The aim of this study was to develop a whole-column imaging-detection capillary isoelectric focusing (icIEF) method for the analytical characterization of charge heterogeneity of a novel humanized anti-EphA2 antibody conjugated to a maytansine derivative. In addition to focusing time, sample composition was optimized: pH range, percent of carrier ampholytes, conjugated antibody concentration, and urea concentration. A good separation of charge isoforms was obtained with 4% carrier ampholytes of a large (3–10) and narrow pH range (8–10.5) (1 : 1 ratio), conjugated antibody concentration (0.3–1 mg/ml) with a good linearity (*R*^2^: 0.9905), 2 M of urea concentration, and 12 minute for focusing. The optimized icIEF method demonstrated a good interday repeatability with RSD values: <1% (pI), <8% (% peak area), and 7% (total peak areas). The optimized icIEF was useful as an analytical characterization tool to assess the charged isoform profile of a discovery batch of the studied maytansinoid-antibody conjugate in comparison to its naked antibody. It exhibited a large pI range (7.5–9.0), while its naked antibody showed a narrow pI range (8.9–9.0). In the discovery batch of maytansinoid-antibody conjugate, 2% of charge isoforms had the same pI as the pI of naked antibody isoforms.

## 1. Introduction

Monoclonal antibodies (mAbs) constitute a very important therapeutic class, with more than 100 mAbs approved by the US Food and Drug Administration (FDA) for the treatment of different diseases [1]. Nearly half of approved mAbs (45%) were anticancer.

Naked mAbs are the most common type used as antitumor. Conjugating antibodies with radioisotopes, chemotherapeutic drugs, or toxins were extensively investigated to improve the antitumor efficiency of an antibody [2–10]. Antibody-drug conjugates (ADCs) can selectively deliver cytotoxic drugs directly to targeted cancer cells.

ADCs may present a considerable heterogeneity resulting from various modifications in the protein structure of antibody itself. Furthermore, linking several drug molecules per antibody decreases the homogeneity of ADC [11, 12]. These probable modifications may lead to the presence of different isoforms in crude and final ADC products. Therefore, full characterization of ADCs should be performed including charge heterogeneity, size heterogeneity, and peptide mapping [13–16].

The main quality attribute of mAb or ADC characterization is the determination of charge variants [17, 18]. Monitoring the charge variants of mAb or ADC gives information on protein stability, product purity from batch to batch, the pathways of degradation, and so on [19].

The charge heterogeneity of ADC is characterized using several analytical methods as well as liquid chromatography [20], ion exchange chromatography (IEC) [21–23], capillary electrophoresis (CE) [24–26], capillary isoelectric focusing (cIEF) [27–32], and imaged capillary isoelectric focusing (icIEF) [33–35].

Isoelectric focusing (IEF) is widely used for the separation of proteins based on their isoelectric point (pI). Compared to conventional gel IEF, cIEF has several advantages. cIEF offers higher resolution, speed, and quantitative analysis [36, 37].

In cIEF, a solution of carrier ampholytes and sample is used to fill the capillary. The two ends of the capillary are placed into acidic and basic solutions. When an electric field is applied, the presence of the ampholytes permits to create a pH gradient through the capillary. Proteins migrate through the capillary until they reach a region of pH equal to their pI, where they become neutral and stop to migrate, resulting in a series of narrow focused zones. In the conventional cIEF, the focalisation of analytes is followed by subsequent mobilization of the focused sample zone to the detection point by different methods (pressure, vacuum, and gravity). Indeed, the mobilization step greatly affects the separation efficiency and reproducibility of the method. Also, the single-point detection cannot permit to monitor the progress of the separation process. Whole-column detection (WCD), for which there is no mobilization step, allows for the simultaneous detection along the entire length of a column and is a better option.

The aim of this study was to separate and determine the pI isoform profile of a novel humanized anti-EphA2 antibody conjugated to a cytotoxic maytansine derivative. To achieve this objective, a whole-column imaging-detection capillary IEF (icIEF) method was optimized and applied to a discovery batch of the studied maytansinoid-antibody conjugate.

## 2. Materials and Methods

### 2.1. Materials

Kit ICE280 chemical test, Kit ICE280 electrolytic solution, methyl cellulose, and 1%, 0.5%, and pI markers (6.6, 7.05, 8.18, 9.5, and 10.10) were purchased from Convergent Bioscience. Urea was from Sigma. Pharmalyte solutions (3–10 and 8–10.5) were from GE healthcare.

A monoclonal naked antibody and its maytansinoid conjugate were analyzed. The discovery batch of antibodies was formulated in HGS buffer consisted of histidine 10 mM, glycine 130 mM, and sucrose 5% (w/v).

### 2.2. Sample Preparation

A protein sample was prepared by diluting to a desired final concentration in 0.35% methyl cellulose, 2% pharmalytes (3–10), and 2% pharmalytes (8–10.5) (1 : 1 ratio) and urea of the desired concentration. pI markers (6.61, 8.81, 7.05, and 9.5) were added to the sample for pI calibration.

An example demonstrating the preparation of an antibody sample (8 mg/ml) (final volume 200 *μ*l and final concentration 1 mg/ml) was reported in Table 1. Each test sample was then vortexed by centrifugation. After centrifugation, the sample was transferred to a glass autosampler vial and centrifuged to remove bubbles before placing in the autosampler carousel for analysis.

### 2.3. icIEF Apparatus

The icIEF analysis was conducted using an iCE280 instrument with a PrinCE autosampler and capillary cartridge from Convergent Bioscience. A transparent capillary column (50 mm, 100 *μ*m ID, and 200 *μ*m OD) is embedded into the glass cartridge, with its inner surface coated with a fluorocarbon to minimize electroosmotic flow. Reservoirs for cathodic (100 mM NaOH and 0.1% methyl cellulose) and anodic solutions (80 mM H_3_PO_4_ and 0.1% methyl cellulose) were attached to the glass cartridge and separated from the capillary by the hollow fiber membrane. Protein focusing time ranged from 7 to 15 min at 3000 V, and detection at 280 nm was achieved with a CCD camera.

## 3. Results and Discussion

### 3.1. Development of the icIEF Method

To characterize the charge heterogeneity of maytansinoid-antibody, the sample, in the preliminary experiments, composed of ADC, 0.35% methyl cellulose, 4% of ampholyte solution with a pH range from 3 to 10 (pharmalytes 3–10), 2 M urea, and two pI markers (6.61 and 9.50).

In order to optimize the separation of charge variants of maytansinoid-antibody by the icIEF method, several experimental parameters have to be considered and optimized: sample pH range and percent of carrier ampholytes, sample ADC concentration, sample urea concentration, and focusing time.  Table 2 summarizes the different experimental conditions for the optimization of charge isoform separation of studied ADC by icIEF.

#### 3.1.1. pH Range of Carrier Ampholytes

**Figure 1:**
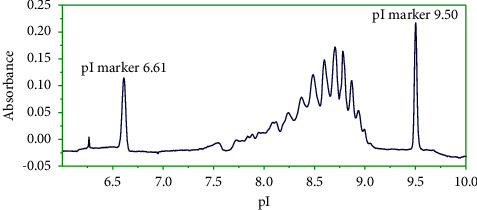
Analysis of maytansinoid-antibody by icIEF. Experimental conditions: final concentration 1 mg/mL in 0.35% methyl cellulose, 2% pharmalytes 3–10 and 2% pharmalytes 8–10.5 (1 : 1 ratio), 2 M urea, pI markers: 6.61, 9.50. Focusing time: 10 min at 3000 V. *λ* = 280 nm.

A solution of carrier ampholytes contains an extensive mixture of zwitterionic compounds of different pI values, which can produce a pH gradient in the capillary. With uniform absorbance along the whole pH range at 280 nm, they are commercially available at different ranges of pH: wide (3–10) and narrow (8–10.5, 5–8). In the preliminary conditions, some charge isoforms were co-migrated with the marker 9.50 using 4% ampholyte solution with a wide pH range (3–10). Therefore, the ampholyte solution with a narrow pH range from 8 to 10.5 (pharmalytes 8–10.5) was added to the pharmalyte solution (3–10) in ratio 1 : 1. Thanks to the addition of ampholytes with narrow pH range, the different charge isoforms of maytansinoid-antibody were separated from the markers 9.5 (Figure 1). As we can see from this figure, the studied maytansinoid-antibody had several isoforms with pI values varied from 7.5 to 9.0.

#### 3.1.2. Maytansinoid-Antibody Concentration

**Figure 2:**
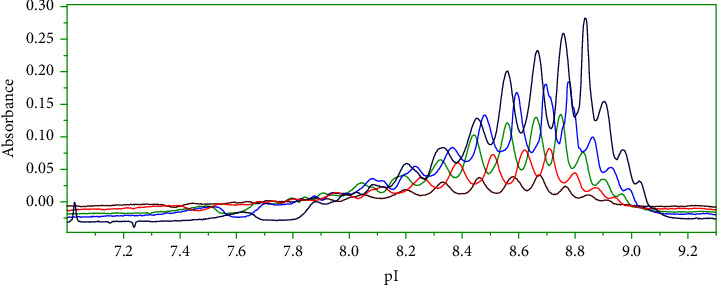
Analysis of different concentrations of maytansinoid-antibody by icIEF (0.3, 0.5, 0.8, 1, and 1.5 mg/ml). Other experimental conditions are as mentioned in Figure 1.

In order to study the effects of the final concentration sample on charge isoform separation, maytansinoid-antibody samples were prepared at different final concentrations: 0.3, 0.5, 0.8, 1, and 1.5 mg/ml (Figure 2).

The increase of maytansinoid-antibody concentrations led to a shift of pI isoforms values towards higher values. This shift of pI values may be related to the increase of additives with increasing maytansinoid-antibody concentration in the sample matrix (histidine, glycine, and sucrose). These additives can impact the linearity of the ampholyte pH gradient and lead to changes of pI values [38]. These additives may also adsorb on the wall of capillary and suppress residual electroosmotic flow, leading to a shift of pI isoforms values.

**Figure 3:**
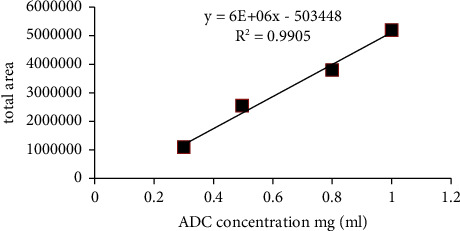
Plot of total peak area against maytansinoid-antibody concentration (0.3, 0.5, 0.8, 1, and 1.5 mg/ml).

A saturation of the UV absorbance was observed at a concentration of maytansinoid-antibody of 1.5 mg/ml. A good linearity was demonstrated for the measurement of isoforms over the range of sample concentrations (0.3–1 mg/ml) (Figure 3). For the following studies, a maytansinoid-antibody concentration of 1 mg/ml was chosen.

#### 3.1.3. Urea Concentration

**Figure 4:**
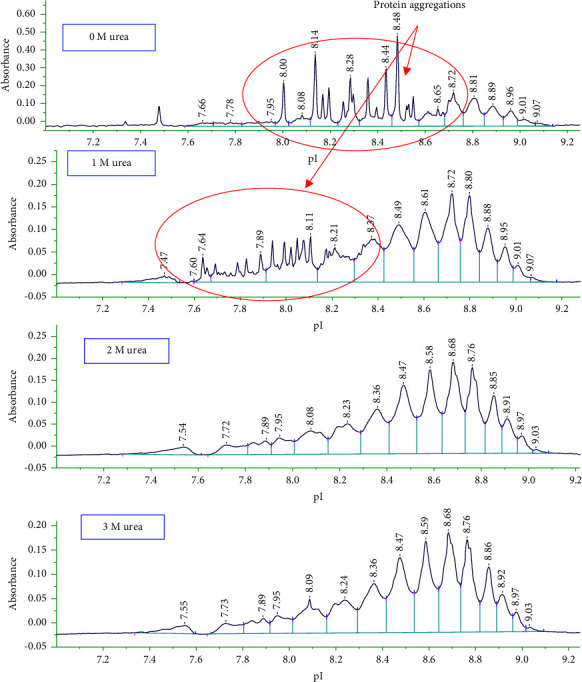
Effects of urea concentration on the separation of charge isoforms of maytansinoid-antibody by icIEF (0, 1, 2, and 3 M urea). Other experimental conditions are as mentioned in Figure 1.

In cIEF, the charge isoform separations can be greatly affected by protein aggregation and precipitation resulted from low protein solubility at or near the pI and at high protein concentrations in the focused bands. Out of a few tested additives known to increase protein solubility, such as nonionic surfactants or urea, the presence of urea had a significant influence on charge isoform separation of ADC. The amount of urea in a sample matrix of maytansinoid-antibody has been varied over the concentration range from 0 to 3 M (Figure 4).

Clearly, the presence of urea improved the charge profile of the maytansinoid-antibody. At a urea concentration of 0 or 1 M, there were the spikes due to the aggregation of proteins into the capillary. However, the charge isoform separation profile was stable over a urea concentration of 2 M. Therefore, 2 M urea has been chosen as the optimal concentration for charge isoform separation of maytansinoid-antibody for the following experiments.

**Figure 5:**
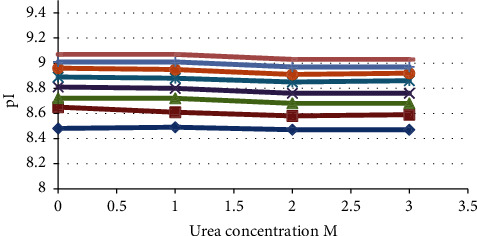
Effect of urea concentration on the pI values of main ADC charge variants.

Urea concentration can affect the separation of protein charge isoforms and the apparent pI values [38, 39]. For example, Turner and Schiel studied the effects of urea concentration on the apparent pI of the main mAb charge variants [38]. The results showed that the apparent pI of the main mAb charge variant was significantly affected by added urea, probably due to urea-mediated denaturation. In order to clarify the impact of added urea, pI values of main ADC charge variants were plotted against urea concentration (Figure 5). A slight decrease of the pI values was remarked with increasing urea concentration.

We should note that urea addition also increased the viscosity of the solution in the capillary and decreased the electrophoretic mobility of the components. Therefore, focusing times need to be optimized for the selected urea concentration.

#### 3.1.4. Focusing Time

**Figure 6:**
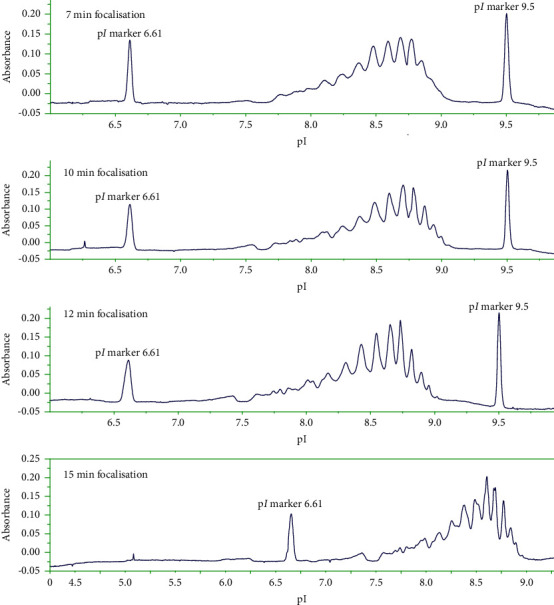
Effects of focusing time on the separation of charge isoforms of maytansinoid-antibody by icIEF. Other experimental conditions are as mentioned in Figure 1.

Focusing time was varied between 7 and 15 min to ascertain that it was long enough for the maytansinoid-antibody isoforms of high relative molecular masses. The profile of maytansinoid-antibody, as shown in Figure 6 as a function of focusing time, indicated that the resolution between charge isoforms improved by increasing the focusing time from 7 min to 15 min. However, the focusing time of 15 min was too long; the marker of pI (9.5) disappeared from the window of detection. Therefore, the focusing time of 12 min was chosen as the optimal conditions of charge isoforms separation.

#### 3.1.5. Ampholyte Percent

**Figure 7:**
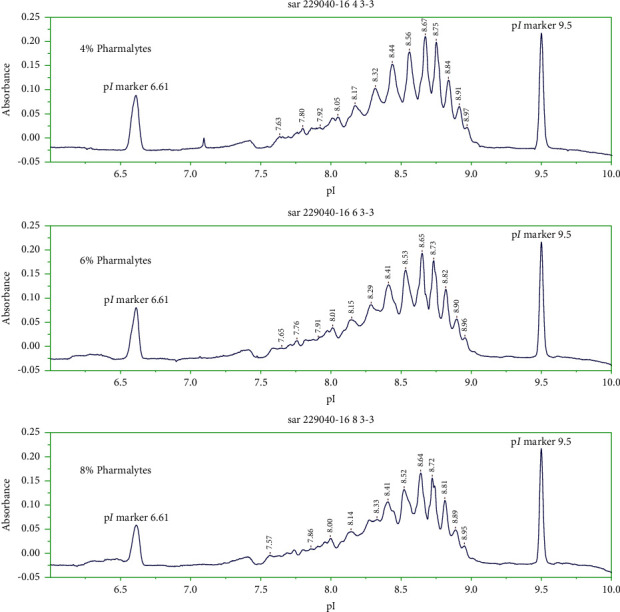
Effects of % pharmalytes on the separation of charge isoforms of maytansinoid-antibody by icIEF. Other experimental conditions are as mentioned in Figure 1.

In order to optimize the separation of charge isoforms of maytansinoid-antibody, the effects of ampholyte percent of 1 : 1 mixture of a narrow pH range (8–10.5) and a wide pH range (3–10) were studied: 4%, 6%, and 8% (Figure 7). As shown in Figure 7, increasing the ampholyte percent led to a decrease of resolution between maytansinoid-antibody isoforms. These results demonstrated that the 4% ampholytes are a good choice for the separation of maytansinoid-antibody charge isoforms.

To develop a method for separation and pI determination of charge isoforms of maytansinoid-antibody by icIEF, different experimental conditions were optimized.

With increasing maytansinoid-antibody concentration in the sample matrix from 0.3 to 1.5 mg/ml, a shift of pI values of charge variants to higher values was observed. In addition, loss of linearity was remarked when increasing maytansinoid-antibody concentration to 1.5 mg/ml as a result of a saturation of UV absorbance. The maytansinoid-antibody concentration was fixed at 1 mg/ml.

The pI markers of 6.61 and 9.50 were the appropriate markers as internal standards since the pI values of charge isoforms of maytansinoid-antibody ranged from 7.5 to 9.0.

The addition of narrow range (8–10.5) to wide range (3–10) pharmalytes led to the improvement of resolution between charge isoforms of maytansinoid-antibody. With increasing the percent of mixture of wide range and narrow range pharmalytes (1 : 1 ratio) from 4% to 8%, the quality of charge isoform separation decreased. Therefore, 4% pharmalytes have been selected to ensure the optimal resolution.

The urea concentration in the sample matrix has been varied from 0 to 3 M. The presence of urea led to the increase of maytansinoid-antibody solubility (the absence of peaks corresponding to antibody aggregates in the icIEF profile) and to improve the resolution of charge isoforms. The urea concentration had slight effects on the charge isoform pI values. The separation of maytansinoid-antibody charge isoforms was stable from 2 to 8 m urea. 2 M urea has been selected to obtain optimal and repeatable charge isoform resolution.

In addition to the different additives in the sample matrix of the maytansinoid-antibody, focusing time is a key parameter to obtain a repeatable and stable icIEF profile. With increasing the focusing time from 7 to 15 min, the resolution of charge isoforms increased. A loss of marker of pI (9.5) was observed at 15 min. Therefore, 12 min at 3000 V was enough time to achieve the optimal separation of charge isoforms.

### 3.2. Interday Repeatability of the Optimized icIEF Method

To test the interday repeatability of the developed method for maytansinoid-antibody, icIEF profiles were performed within three consecutive days. The statistically obtained results, summarized in Table 3, demonstrated a good interday repeatability.

### 3.3. Application of the Developed icIEF Method

**Figure 8:**
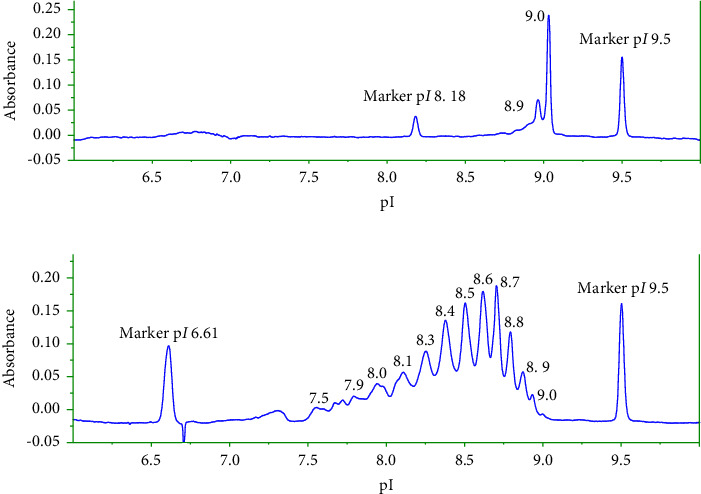
icIEF profiles of naked antibody (a) and its corresponding maytansinoid conjugate (b). Final concentration of maytansinoid-antibody in sample matrix is 1 mg/ml diluted in 0.35% methyl cellulose, 4% 3–10 pharmalytes/8–10.5 pharmalytes (1 : 1 ratio), and 2 M urea, pI markers: 8. 18, 9.50 (a) and 6.61, 9.50 (b), and focusing time of 12 min at 3000 V. Other experimental conditions are as mentioned in Figure 1.

The developed icIEF method was applied to characterize the charge heterogeneity profile of the discovery batch of the studied maytansinoid conjugate (Figure 8(a)) in comparison with its naked antibody (Figure 8(b)). The naked antibody showed a narrow pI range (8.9–9.0), while the studied maytansinoid conjugate exhibited a large pI range (7.5–9.0). The level of unconjugated antibody in an ADC formulation is a critical parameter in process control because it can directly affect the efficacy of ADC. From Table 4, the percent of charge isoforms corresponding to naked antibody represented 2% of charge isoforms in the ADC discovery batch. These results demonstrated the success of the conjugating process of a naked antibody with a maytansinoid derivative.

This heterogeneity of ADC is related to the covalent linking of the cytotoxic drug to the free amine groups of lysine of mAb. Monoclonal antibodies often have 40–60 lysine residues, and chemical conjugation results in a heterogeneous mixture consisting of unconjugated mAbs and mAbs conjugated with a variable number of cytotoxins in random combinations at different sites on the antibody [40, 41]. The charge variants of maytansinoid conjugate differed by the number of amine groups of lysine conjugated to a linker molecule, leading to decrease their pI with increase of the number of modified amino groups (more acidic). The obtained results were in agreement with a study of charge variants of IgG1-Fc and conjugated IgG1-Fc performed by Boylon et al. [42]. The study demonstrated that the chemical conjugation of IgG1-Fc to different drugs via the amino acid Lys modified the electrostatic properties of the mAb surface and introduces further complexities. The chemical conjugation led to the decrease in pI upon conjugation.

The developed chromatofocusing is suitable for the analytical characterization of charge heterogeneity of the studied monoclonal antibody and its drug conjugate based on differences in the isoelectric points of the variants presents.

## 4. Conclusion

Whole-column imaging-detection capillary isoelectric focusing (icIEF) is a promising technique to check the quality of therapeutic proteins. In this work, an icIEF method was developed for monitoring the charge heterogeneity of maytansinoid-monoclonal antibody. The composition of maytansinoid-monoclonal antibody was optimized including pH and percent of carrier ampholytes, conjugated antibody concentration, urea concentration, and focusing time. Under optimized condition, peaks corresponding to charge variants of conjugated antibody were separated with good resolution. Method results showed a good interday repeatability and linearity within the concentration range of 0.3–1 mg/ml. The developed method was applied to assess the quality of discovery batch of maytansinoid-monoclonal antibody [43].

## Figures and Tables

**Table 1:** An example of 200 *μ*l sample preparation of ADC sample.

1 mg/ml	Volume *μ*l	Final concentration
Methyl cellulose 1%	70	0.35%
Pharmalytes 3–10	4	2%
Pharmalytes 8–10.5	4	2%
Urea (8 M)	50	2 M
ADC 8 mg/ml	25	1 mg/ml
Water Milli-*Q*	45	
pI marker 6.61	1	
pI marker 9.5	1	

**Table 2:** Optimized experimental parameters for ADC analyses by iCE280.

Ampholyte pH range	Pharmalytes 8–10.5/pharmalytes 3–10
Sample concentration	0.3, 0.5, 0.8, 1, and 1.5 mg/ml
Urea concentration	0, 1, 2, and 3 M
Focusing time	7, 10, 12, and 15 min
% ampholytes	4%, 6%, and 8%

**Table 3:** Statistical results of interday repeatability of icIEF profile of maytansinoid-antibody (*n* = 6 and 3 days).

pI			% area			Total area		
Mean	SD	RSD%	Mean	SD	RSD%	Mean	SD	RSD%
8.4	0.017	0.2	11	0.440	4	524000	36680	7
8.5	0.034	0.4	15	0.750	5			
8.6	0.017	0.2	13	0.650	5			
8.7	0.026	0.3	11	0.770	7			
8.8	0.009	0.1	7	0.280	4			
8.9	0.009	0.1	4	0.160	4			
9	0.018	0.2	1.7	0.102	6			

**Table 4:** Comparison of charge profiles of studied naked antibody and its corresponding ADC, including isoform number, pI range, ΔpI, and pI and % area of major species.

	Isoform number	pI range	ΔpI	pI and % area of major species
Naked antibody	2	8.9–9.0	0.1	Peak 1: pI 8.9 : 36%, Peak 2: pI 9.0 : 64%
Maytansinoid-antibody	12	7.5–9	1.5	Peak 1: pI 8.4 : 11%, Peak 2: pI 8.5 : 15%, Peak 3: pI 8.6 : 13%, Peak 3: pI 8.7 : 11%, Peak 4: pI 8.8 : 7%, Peak 5: pI 8.9 : 4%, Peak 6: pI 9.0 : 1.7%

## Data Availability

The data used to support the findings of this study are available from the corresponding author upon request.
